# Physical activity and COVID-19: an observational and Mendelian randomisation study

**DOI:** 10.7189/jogh-10-020514

**Published:** 2020-12

**Authors:** Xiaomeng Zhang, Xue Li, Ziwen Sun, Yazhou He, Wei Xu, Harry Campbell, Malcolm G Dunlop, Maria Timofeeva, Evropi Theodoratou

**Affiliations:** 1 Centre for Global Health, Usher Institute, The University of Edinburgh, Edinburgh, UK; 2 School of Public Health and the Second Affiliated Hospital, Zhejiang University, Hangzhou, China; 3 School of Design and Arts, Beijing Institute of Technology, Beijing, China; 4 Edinburgh School of Architecture and Landscape Architecture, The University of Edinburgh, Edinburgh, UK; 5 Colon Cancer Genetics Group, Cancer Research UK Edinburgh Centre and Medical Research Council Human Genetics Unit, Medical Research Council Institute of Genetics and Molecular Medicine, The University of Edinburgh, Edinburgh, United Kingdom; 6 DIAS, Danish Institute for Advanced Study, Department of Public Health, University of Southern Denmark, Odense, Denmark; 7 Cancer Research UK Edinburgh Centre, MRC Institute of Genetics and Molecular Medicine, The University of Edinburgh, Edinburgh, UK

## Abstract

**Background:**

Physical activity (PA) is known to be a protective lifestyle factor against several non-communicable diseases while its impact on infectious diseases, including Coronavirus Disease 2019 (COVID-19) is not as clear.

**Methods:**

We performed univariate and multivariate logistic regression to identify associations between both objectively and subjectively measured PA collected prospectively and COVID-19 related outcomes (overall COVID-19, inpatient COVID-19, outpatient COVID-19, and COVID-19 death) in the UK Biobank cohort. Subsequently, we tested causality by using Mendelian randomisation (MR) analyses.

**Results:**

In the multivariable model, the increased acceleration vector magnitude PA (AMPA) is associated with a decreased probability of overall and outpatient COVID-19 with an odds ratio (OR) and 95% confidence interval (CI) of 0.80 (0.69, 0.93) and 0.74 (0.58, 0.95), respectively. No association is found between self-reported moderate-to-vigorous PA (MVPA) and COVID-19 related outcomes. No association is found by MR analyses.

**Conclusions:**

Our results indicate a protective effect of objectively measured PA and COVID-19 outcomes (outpatient COVID-19 and overall COVID-19) independent of age, sex, measures of obesity, and smoking status. Although the MR analyses do not support a causal association, that may be due to limited power. We conclude that policies to encourage and facilitate exercise at a population level during the pandemic should be considered.

Coronavirus Disease 2019 (COVID-19) caused by severe acute respiratory syndrome coronavirus 2 (SARS-CoV-2) was declared a pandemic on March 11, 2020, by the World Health Organisation (WHO). By October 16th, 2020, a total of 39 023 292 patients had been diagnosed, with 1 099 586 confirmed deaths globally [1]. National responses to the pandemic including a range of non-pharmacological interventions (NPIs) have been adopted by countries at nation-wide, state-wide, or city-wide levels. The restrictions imposed by governments during the outbreak have had a substantial impact on patterns of physical activity (PA) within populations. For example, an online survey conducted in Canada indicated that 40.5% of physically inactive individuals became less active and 33% became more active; 22.4% of physically active individuals became less active and 40.3% became more active during lockdown time [2].

PA is known to be a protective lifestyle factor for a number of non-communicable diseases (eg, cancer and cardiovascular disease) and ageing processes (eg, immunosenescence) [3-5]. However, evidence of the role of PA on respiratory viral infections remains weak, especially for highly contagious viruses like SARS-CoV-2. Since doing exercise could compromise social distancing measures and increase opportunities for contracting the virus (probably indoors more than outdoors), it is unclear whether being physically active is a beneficial lifestyle factor for respiratory viral infections. Effects of PA on COVID-19 could be confounded by the effects of obesity. Viral pathogenesis has been reported to be greater in obese or overweight individuals and obesity has been suggested as a risk factor for COVID-19 [6,7]. In this study, we analysed whether PA influences the risk of COVID-19 in a prospective observational study by adjusting measures of obesity and smoking status. To avoid possible confounding effects induced by observational studies, we further investigated any associations between genetic predisposition of PA and COVID-19 by applying Mendelian Randomisation (MR) analyses.

## MATERIALS AND METHODS

### Data set

The UK Biobank (UKBB) is a prospective cohort study including more than 500 000 participants aged from 40 to 69 years in the United Kingdom. In this study, participants who have not been tested positive for SARS-CoV-2 and not died of COVID-19 were taken as controls. We removed from the controls the following participants in a sensitivity analysis: i) Those who tested negative since test results could have been false negative; ii) Participants, who were not from England, since all COVID-19 test results were provided by NHS England only; iii) Participants who died before 01/01/2020. We used both self-reported moderate-to-vigorous PA (MVPA) and acceleration vector magnitude PA (AMPA) as measures of PA. We took body mass index (BMI), waist circumference and hip circumference as measures of obesity in the prospective observational study. The detail of MVPA, AMPA and measures of obesity data were described in the supplementary method.

UKBB released COVID-19 test results with the date of the specimen taken, specimen type (eg, nasal and throat), the testing laboratory, whether the patient was an inpatient or outpatient when the sample was taken and SARS-CoV-2 testing results. Inpatient samples included inpatient infections, samples taken in emergency departments and health care related infections. From 16/03/2020 to 29/06/2020, there were 14 439 SARS-CoV-2 test results among UKBB participants with 1596 participants having at least one SARS-CoV-2 positive result and 7898 participants having one or more negative results. Of these individuals, 7187 were inpatients and 2307 were outpatients. We analysed four COVID-19 related outcomes: overall COVID-19, included all patients (inpatients, outpatients or deaths); inpatient COVID-19, included all inpatients with at least one positive SARS-CoV-2 testing result; outpatient COVID-19, included outpatients who tested SARS-CoV-2 positive at least once; COVID-19 death, included death caused by clinical and epidemiological diagnosed COVID-19 (both primary and contributory causes of death).

Subsequently, we performed MR analyses to test the causality between PA and COVID-19 in the UKBB. The detailed description of genotyping, quality control and genetic imputation have been described previously [8]. From a total of 488 366 participants in the UKBB with genotype data, 149 110 samples were excluded due to consent withdrawals, non-white British ethnic background, sex mismatch, sex aneuploidy, high missing rate/outlier, and kinship inference. The genetic instruments for MVPA and AMPA were extracted from the largest available genome-wide association study (GWAS) data sets [9,10]. Seven and five common genetic variants were extracted for MVPA and AMPA respectively after considering linkage disequilibrium (LD) (r^2^>0.2). Then, we performed multivariable MR to adjust BMI. The effect estimates of PA SNPs on BMI were extracted from a BMI GWAS [11].

### Statistical analyses

To describe the characteristics of participants, mean and standard deviation (SD) were presented for continuous covariates, and number (N) and percentage (%) were presented for categorical covariates. We performed both univariate and multivariate logistic regression analyses to test the association between two measures of PA and four COVID-19 related outcomes. In the multivariate logistic regression model, we first added age and sex as covariates, then we further added measures of obesity or overweight (waist circumference, hip circumference, and BMI), and finally, we added smoking measures (smoking status, exposure to smoking at home and exposure to smoking out of home). The details of the covariates can be found in Bycroft et al [12]. Since measures of obesity or overweight tend to be correlated, we analysed the Pearson correlation coefficients among BMI, waist circumference and hip circumference. Table S1 in the **Online Supplementary Document** shows that BMI, waist circumference and hip circumference are significantly correlated, but the correlation coefficients are less than 0.1. Multicollinearity would not be an issue in our multivariable models.

For the MR analysis, we tested the association between genetic instruments of the exposures and COVID-19 by performing multivariate logistic regression adjusting for age, sex, the first 10 principal components (PCs), and the assessment centre in UKBB. The causal effects and the corresponding standard errors of exposures on the outcome were calculated by using a random effect inverse-variant weighted (IVW) method [13]. We evaluated the heterogeneity among the causal effects of each variant (Cochran’s Q statistic) and a *P* less than 0.10 was regarded as statistically significant heterogeneity. We performed MR-Egger [14] as a sensitivity analysis to explore the potential bias introduced by horizontal pleiotropy.

The *P* threshold is set at 0.05 for all the analyses. All statistical analyses were performed on R v3.6.1 (Foundation for Statistical Computing, Vienna, Austria).

## RESULTS

**Figure 1:**
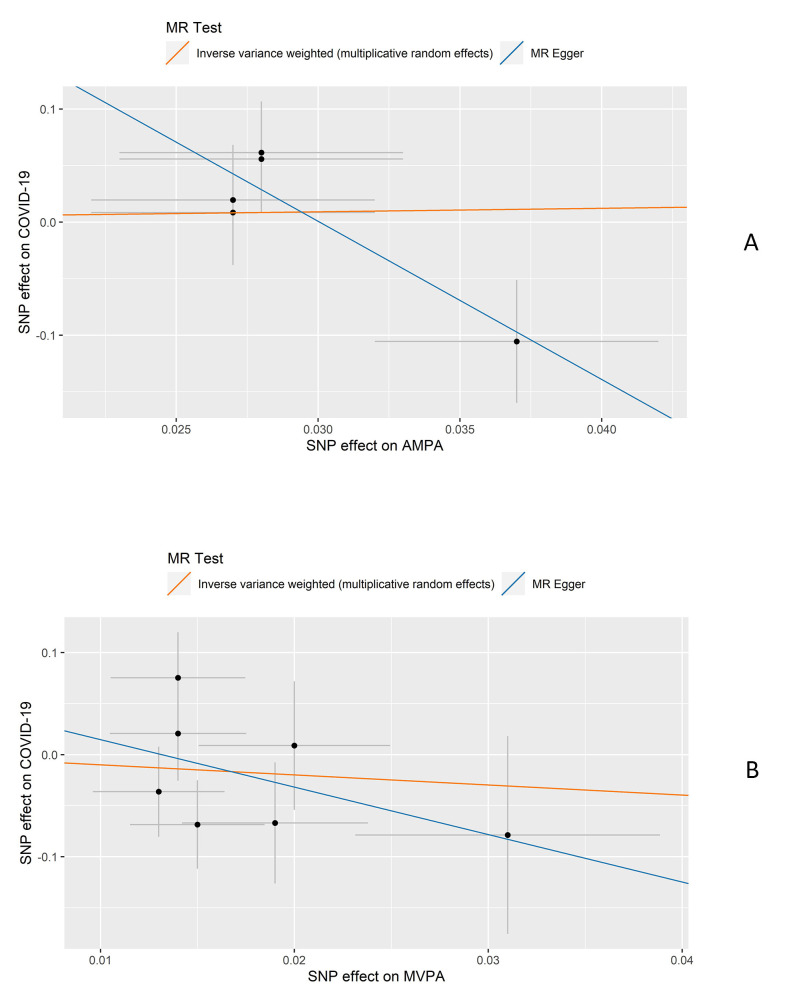
Scatter plots of results from Mendelian randomisation (MR) analysis. **Panel A.** Scatter plot of AMPA on COVID-19. **Panel B.** Scatter plot of MVPA on COVID-19. MVPA – moderate-to-vigorous physical activity, COVID-19 – coronavirus disease 2019, AMPA – acceleration vector magnitude physical activity.

The characteristics of participants in the four outcome groups (overall COVID-19, inpatient COVID-19, outpatient COVID-19, and COVID-19 death), and the included controls are described in **Table 1**. Patients who died from COVID-19 had the highest average age (74.7) while outpatients had the lowest average age (66.0). The proportion of males was the highest for the COVID-19 death group (63.9%) and the lowest for the outpatient COVID-19 group (45.8%). Participants in the inpatient COVID-19 group had the longest MVPA time while participants in the COVID-19 death group had the shortest. For AMPA, participants in the control group had the longest AMPA time while patients who died from COVID-19 had the shortest. The results of univariate and multivariate logistic regression of two measures of PA on the four outcomes are presented in **Table 2**. The results of MR analyses are presented in **Figure 1** and **Table 3**.

**Table 1:** Characteristics of UK Biobank participants

	Overall COVID-19 cases	Inpatient COVID-19 cases	Outpatient COVID-19 cases	COVID-19 deaths	Controls	Controls*
	**mean (SD)/n (%)**	**mean (SD)/n (%)**	**mean (SD)/n (%)**	**mean (SD)/n (%)**	**mean (SD)/n (%)**	**mean (SD)/n (%)**
All participants	1746 (0.4%)	1020 (0.2%)	576 (0.1%)	399 (0.1%)	500 758 (99.7%)	415 596 (99.7%)
Age	68.8 (9.2)	69.4 (8.9)	66.0 (9.4)	74.7 (6.0)	68.5 (8.1)	68.1 (8.1)
Sex:						
Male	924 (52.9%)	570 (55.9%)	264 (45.8%)	255 (63.9%)	268 549 (54.5%)	185 494 (44.6%)
Female	822 (47.1%)	450 (44.1%)	312 (54.2%)	144 (36.1%)	224 485 (45.5%)	230 102 (55.4%)
MVPA (MET-minutes/week)	990.4 (1310.8)	1039.0 (1356.1)	898.7 (1236.7)	1017.0 (1057.9)	973.8 (1269.0)	974.6 (1268.1)
AMPA (milli-gravities)	26.7 (8.6)/(n = 215)	26.6 (8.9)/(n = 122)	27.4 (8.1)/(n = 79)	24.1 (8.1)/(n = 36)	28.0 (8.2)/(n = 96 460)	28.1 (8.2)/(n = 83 748)
BMI (kg/m^2^)	27.5 (4.9)	27.5 (4.8)	27.7 (5.0)	27.6 (4.7)	27.4 (4.8)	27.4 (4.8)
Waist circumference (cm)	90.8 (14.0)	91.3 (14.5)	90.2 (13.2)	90.7 (14.6)	90.3 (13.5)	90.3 (13.5)
Hip circumference (cm)	103.1 (9.5)	102.9 (9.8)	103.4 (8.8)	103.2 (9.3)	103.4 (9.3)	103.4 (9.2)
Smoking status:						
Never	780 (48.9%)	462 (45.3%)	318 (55.2%)	148 (37.1%)	269 014 (54.5%)	231 192 (55.6%)
Previous	619 (38.8%)	429 (42.0%)	190 (33.0%)	186 (46.6%)	169 392 (34.3%)	140 876 (33.9%)
Current	179 (11.2%)	116 (11.4%)	63 (10.9%)	60 (15.0%)	51 748 (10.5%)	41 182 (9.9%)
Unknown	18 (1.1%)	13 (1.3%)	5 (0.9%)	5 (1.3%)	3880 (0.8%)	1595 (0.6%)
Exposure to smoking at home (hour/week)	0.5 (4.1)	0.5 (4.5)	0.5 (3.8)	0.4 (4.0)	0.5 (4.4)	0.5 (4.5)
Exposure to smoking out of home (hour/week)	0.4 (2.3)	0.4 (2.3)	0.4 (2.4)	0.4 (3.4)	0.4 (2.4)	0.4 (2.5)

COVID-19 – coronavirus disease 2019, N – number, SD – standard deviation, BMI – body mass index, MVPA – self-reported moderate-to-vigorous physical activity, AMPA – acceleration vector magnitude physical activity

*A control group that excludes participants who have tested negative for SARS-CoV-2, who are not in England, and who died before 1 January 2020.

**Table 2:** Association analyses between measures of physical activity and four COVID-19 related outcomes

	Overall COVID-19				Inpatients COVID-19				Outpatients COVID-19				COVID-19 death			
	**MVPA (subjectively measured)**		**AMPA (objectively measured)**		**MVPA (subjectively measured)**		**AMPA (objectively measured)**		**MVPA (subjectively measured)**		**AMPA (objectively measured)**		**MVPA (subjectively measured)**		**AMPA (objectively measured)**	
	**OR (95%CI)**	***P*-value**	**OR (95%CI)**	***P*-value**	**OR (95%CI)**	***P*-value**	**OR (95%CI)**	***P*-value**	**OR (95%CI)**	***P*-value**	**OR (95%CI)**	***P*-value**	**OR (95%CI)**	***P*-value**	**OR (95%CI)**	***P*-value**
**Univariate logistic regression**																
Physical activity	1.01 (0.97, 1.06)	0.584	0.84 (0.73, 0.98)	**0.021**	1.05 (0.99, 1.11)	0.104	0.83 (0.68, 1.00)	0.056	0.94 (0.86, 1.02)	0.156	0.92 (0.74, 1.16)	0.495	1.03 (0.94, 1.14)	0.493	0.56 (0.38, 0.83)	**0.004**
**Multivariate logistic regression adjusting age and sex**																
Physical activity	1.01 (0.97, 1.06)	0.591	0.80 (0.69, 0.92)	**0.003**	1.05 (0.99, 1.11)	0.105	0.84 (0.69, 1.02)	0.086	0.96 (0.86, 1.02)	0.152	0.74 (0.58, 0.95)	**0.017**	1.03 (0.94, 1.14)	0.485	0.79 (0.53, 1.17)	0.239
Age	1.00 (1.00 1.01)	0.155	0.97 (0.95, 0.99)	3.09×10^-4^	1.01 (1.01, 1.02)	3.70×10^-4^	1.00 (0.97, 1.02)	0.754	0.96 (0.95, 0.97)	2.81×10^-13^	0.90 (0.88, 0.93)	1.46×10^-11^	1.13 (1.11, 1.15)	3.47×10^-45^	1.18 (1.10, 1.26)	2.42×10^-6^
Sex:																
Male	1.34 (1.22, 1.47)	1.09×10^-9^	1.36 (1.04, 1.78)	0.024	1.50 (1.33, 1.70)	1.04×10^-10^	1.83 (1.27, 2.62)	0.001	1.02 (0.87, 1.21)	0.784	0.84 (0.54, 1.33)	0.468	2.00 (1.63, 2.45)	3.41×10^-11^	1.84 (0.93, 3.65)	0.08
**Multivariate logistic regression adjusting age, sex, and measures of obesity**																
Physical activity	1.02 (0.97, 1.06)	0.504	0.80 (0.69, 0.93)	***0.003***	1.05 (0.99, 1.11)	0.108	0.85 (0.70, 1.03)	0.096	0.95 (0.87, 1.03)	0.233	0.74 (0.58, 0.95)	***0.019***	1.04 (0.94, 1.14)	0.449	0.78 (0.52, 1.17)	0.235
Age	1.00 (1.00, 1.01)	0.156	0.97 (0.95, 0.99)	2.66×10^-4^	1.01 (1.01, 1.02)	4.20×10^-4^	1.00 (0.97, 1.02)	0.756	0.96 (0.95, 0.97)	2.51×10^-13^	0.90 (0.87, 0.93)	7.48×10^-12^	1.13 (1.11, 1.15)	3.30×10^-45^	1.18 (1.10, 1.26)	2.45×10^-6^
Sex:																
Male	1.33 (1.21, 1.47)	2.29×10^-9^	1.33 (1.02, 1.75)	0.037	1.50 (1.32, 1.70)	1.70×10^-10^	1.80 (1.25, 2.58)	0.002	1.01 (0.86, 1.19)	0.882	0.80 (0.50, 1.28)	0.351	1.98 (1.61, 2.43)	6.89×10^-11^	1.84 (0.93, 3.65)	0.082
Waist circumference	1.00 (1.00, 1.01)	0.231	1.00 (0.99, 1.01)	0.557	1.00 (1.00, 1.01)	0.05	1.01 (1.00, 1.03)	0.05	1.00 (0.99, 1.01)	0.789	0.99 (0.97, 1.00)	0.097	1.00 (0.99, 1.01)	0.78	1.00 (0.98, 1.03)	0.891
Hip circumference	1.00 (0.99, 1.00)	0.166	1.00 (0.99, 1.02)	0.871	0.99 (0.99, 1.00)	0.101	1.01 (0.98, 1.02)	0.87	1.00 (0.99, 1.01)	0.993	1.00 (0.97, 1.02)	0.884	1.00 (0.99, 1.01)	0.652	0.99 (0.95, 1.03)	0.644
BMI	1.01 (1.00, 1.01)	0.315	1.01 (0.98, 1.04)	0.52	1.00 (0.99, 1.02)	0.67	1.00 (0.96, 1.04)	0.981	1.01 (0.99, 1.03)	0.264	1.03 (0.98, 1.07)	0.21	1.01 (0.99, 1.03)	0.505	0.99 (0.92, 1.06)	0.761
**Multivariate logistic regression adjusting age, sex, measures of obesity and smoking measures**																
Physical activity	1.01 (0.95, 1.06)	0.876	0.82 (0.69, 0.96)	***0.012***	1.05 (0.98, 1.11)	0.166	0.85 (0.69, 1.05)	0.129	0.93 (0.85, 1.03)	0.155	0.79 (0.61, 1.03)	0.078	1.00 (0.90, 1.11)	0.966	0.74 (0.48, 1.14)	0.172
Age	1.00 (1.00, 1.00)	0.144	0.98 (0.96, 0.99)	0.01	1.02 (1.01, 1.02)	4.31×10^-4^	1.01 (0.98, 1.03)	0.658	0.96 (0.95, 0.97)	2.36×10^-12^	0.90 (0.87, 0.93)	8.53×10^-10^	1.13 (1.11, 1.16)	4.46×10^-40^	1.21 (1.12, 1.31)	1.30×10^-6^
Sex:																
Male	1.36 (1.22, 1.50)	5.93×10^-9^	1.41 (1.05, 1.88)	0.021	1.50 (1.31, 1.72)	2.98×10^-9^	1.94 (1.31, 2.88)	9.22×10^-4^	1.08 (0.91, 1.30)	0.373	0.84 (0.51, 1.39)	0.505	1.90 (1.52, 2.37)	1.26×10^-8^	1.57 (0.78, 3.18)	0.207
Waist circumference	1.00 (1.00, 1.01)	0.086	1.01 (1.00, 1.02)	0.133	1.01 (1.00, 1.01)	0.013	1.01 (1.00, 1.03)	0.033	1.00 (0.99, 1.01)	0.747	1.00 (0.98, 1.01)	0.723	1.00 (0.99, 1.01)	0.56	1.00 (0.98, 1.03)	0.93
Hip circumference	1.00 (0.99, 1.00)	0.127	1.00 (0.98, 1.01)	0.793	0.99 (0.99, 1.00)	0.076	1.00 (0.98, 1.02)	0.775	1.00 (0.99, 1.01)	0.962	0.98 (0.96, 1.01)	0.234	1.00 (0.99, 1.01)	0.916	1.00 (0.97, 1.04)	0.821
BMI	1.00 (0.99, 1.01)	0.449	1.01 (0.98, 1.04)	0.375	1.00 (0.99, 1.02)	0.758	1.01 (0.98, 1.05)	0.456	1.01 (0.99, 1.02)	0.486	1.01 (0.97, 1.06)	0.574	1.01 (0.98, 1.03)	0.594	1.01 (0.94, 1.08)	0.844
Smoking status:																
Never (Reference)	1	/	1	/	1	/	1	/	1	/	1	/	1	/	1	/
Previous	1.25 (1.12, 1.40)	7.36×10-^5^	1.38 (1.01, 1.89)	0.041	1.38 (1.19, 1.59)	1.54×10^-5^	1.57 (1.04, 2.36)	0.030	1.01 (0.83, 1.24)	0.895	0.98 (0.57, 1.71)	0.953	1.64 (1.29, 2.08)	4.75×10^-5^	1.93 (0.91, 4.12)	0.089
Current	1.25 (1.06, 1.48)	0.008	1.76 (1.09, 2.84)	0.021	1.23 (0.99, 1.54)	0.067	1.95 (1.03, 3.69)	0.041	1.09 (0.82, 1.45)	0.566	1.51 (0.70, 3.25)	0.291	2.60 (1.87, 3.61)	1.34×10^-8^	3.37 (1.06, 10.73)	0.040
Exposure to smoking at home	1.00 (0.98, 1.01)	0.513	0.95 (0.85, 1.05)	0.291	0.99 (0.97, 1.01)	0.368	0.94 (0.82, 1.09)	0.424	1.00 (0.99, 1.02)	0.647	0.96 (0.82, 1.11)	0.559	1.00 (0.97, 1.02)	0.879	0.65 (0.29, 1.45)	0.293
Exposure to smoking out of home	1.00 (0.98, 1.02)	0.908	0.91 (0.79, 1.05)	0.192	1.00 (0.97, 1.03)	0.962	0.89 (0.73, 1.09)	0.276	1.00 (0.97, 1.04)	0.799	0.80 (0.58, 1.10)	0.169	1.01 (0.97, 1.05)	0.671	1.00 (0.87, 1.15)	0.987

MVPA – self-reported moderate-to-vigorous physical activity, AMPA – acceleration vector magnitude physical activity, OR – odds ratio, CI – confidence interval

**Table 3:** Results of Mendelian randomisation studies between physical activity exposures and overall COVID-19

	MVPA				AMPA			
	**OR (95%CI)**	**P**	**P_int_**	**P_het_**	**OR (95%CI)**	**P**	**P_int_**	**P_het_**
**One-sample Mendelian randomisation:**								
IVW	0.37 (0.03, 5.08)	0.46	/	0.28	1.36 (0.20, 9.21)	0.76	/	0.13
MR-Egger	0.01 (8.58×10^-8^, 1061.38)	0.43	0.53	/	8.42×10^-7^ (4.56×10^-12^, 0.16)	0.02	0.02	/
**Multivariable Mendelian randomisation adjusted BMI:**								
IVW	0.39 (0.02, 6.92)	0.52	/	0.19	0.73 (0.12, 4.25)	0.73	/	0.3
MR-Egger	0.002 (1.60×10^-9^, 1735.41)	0.37	0.43	/	7.66×10^-6^ (2.04×10^-11^, 2.88)	0.07	0.08	/

COVID-19 – coronavirus disease 2019, MVPA – self-reported moderate-to-vigorous physical activity, AMPA – acceleration vector magnitude physical activity, BMI – body mass index, OR – odds ratio, CI – confidence interval, IVW – inverse variance weighted method, P_int_ – *P* value for the intercept of MR-Egger’s test, P_het_ – *P* values of χ2 Q test for heterogeneity.

A total of 500 758 participants (96 460 participants have AMPA data) from the UKBB were included as controls of the observational analyses. For the COVID-19 test result records, 1596 patients were COVID-19 positive, of these, 1020 were inpatients and 576 were outpatients. In addition, 399 participants had died of COVID-19 (with 376 deaths having COVID-19 as the primary cause of death), in which 249 had SARS-CoV-2 positive results. In the multivariate logistic regression models, AMPA is associated with decreased risk of contracting COVID-19 and attending as an outpatient with a COVID-19 related health concern. The odds ratio (OR) and 95% confidence interval (95% CI) per SD increase of AMPA are 0.80 (0.69, 0.93) and 0.74 (0.58, 0.95) respectively after adjusting for age, gender, and measures of body fatness (**Table 2**). Further adjusting smoking status, the OR (95%CI) is 0.81 (0.69, 0.96) for the association between AMPA on overall COVID-19. In the univariate model, AMPA relate to a decreased risk of COVID-19 death while the association disappeared after adjusting for the other covariates. On the contrary, MVPA is not associated with any of the COVID-19 outcomes (**Table 2**). The sensitivity analyses report similar results with the main analyses (Table S2 in the **Online Supplementary Document**).

A total of 342 678 participants with genotype data were included in the MR analyses after sample quality control. Of these, 9494 participants had COVID-19 test results. The effect estimates of the instrumental variables on COVID-19 and BMI are listed in Table S3 in the **Online Supplementary Document**. AMPA detect to be associated with a lower risk of inpatient COVID-19 by the MR-Egger method in the one-sample MR, however, the intercept of MR-Egger indicates the existence of pleiotropic effects. Significant heterogeneity has not been detected by Q statistics (**Figure 1** and **Table 3**). MVPA is not found to be causally linked to COVID-19 outcomes. After adjusting BMI in the multivariable MR, no association is indicated.

## DISCUSSION

Our study found that higher AMPA value was inversely associated with overall COVID-19 (1746 cases) and outpatient COVID-19 (576 cases) after adjusting for age, sex, measures of obesity in the multivariate model. The association between AMPA and overall COVID-19 persisted after adjusting for smoking status. No association was detected by observational analyses between MVPA and COVID-19. No causal association was found between any measure of PA and COVID-19 outcomes in the MR analyses.

PA can benefit respiratory viral infections through increasing the endurance of the respiratory muscles or improving the immune response to respiratory viral antigens [15], however, the direct mechanism is unclear. One review reported that moderate-intensity exercise reduces the risk and severity of respiratory viral infections while vigorous-intensity exercise increased the risk of self-reported respiratory viral infection symptoms [16]. Since there was limited testing capacity at the early stage of the pandemic in the UK, most (76%) of the SARS-CoV-2 testing samples came from hospitalised patients, which could be taken as a surrogate for comparably serious disease associated with COVID-19 [17]. In our study, overall COVID-19 covered confirmed cases from inpatient and outpatient settings, and patients, who died of or with COVID-19. Inpatient COVID-19 indicated patients with serious COVID-19 while outpatient COVID-19 indicated patients with relatively milder symptoms. In our analysis, one SD (8.14 milli-gravities) increase of AMPA approximated to 3MET-hour/d [18], which meant if an individual could replace 55 minus sedentary behaviour with hiking daily, the COVID-19 risk could decrease around 20%. AMPA related to a decreased risk of overall COVID-19 and outpatient COVID-19 but not inpatient COVID-19 and COVID-19 death. The effect estimates implied that the objectively measured PA had a larger effect on relatively mild COVID-19 outcomes. The effect of AMPA was abrogated after adjustment for other covariates, which highlighted the importance of the effect of age, gender, and other co-morbidities for COVID-19 death.

Published evidence shows that PA was not only associated with better physical health through pathways such as regulating immunity [19] but also could benefit mental health [20]. There is a possibility that the higher PA might be a proxy for better biological or mental health. Meanwhile, PA is associated with better socio-economic status and other healthy behaviours (eg, diet) [21] which may indicate a better adhesion to social distancing rules or using of personal protective equipment during a pandemic.

This is the first study to analyse the association between PA and COVID-19 quantitatively. One of the advantages of this study is that both objectively and subjectively measured PA were analysed. AMPA is widely considered as a relatively accurate measure of PA, whereas the main advantage of using MVPA is its cost-efficiency. MVPA and AMPA measures were compared in a large Norwegian study and the results indicated that people tend to report less moderate-intensity and more vigorous-intensity PA compared to AMPA regardless of gender and age [22]. Older adults also tended to exaggerate the reports of MVPA [22], perhaps because fragile individuals experienced stronger proprioception during exercise. In addition, self-reported questionnaires tended to record more intentional outdoor exercise while accelerometers measure all types of exercise [18,23]. During the pandemic, these differences may intensify. Another advantage of this study is that four COVID-19 outcomes were analysed. Although more data reflecting serious diseases related to COVID-19 is available, the four outcomes can still represent different severity of COVID-19 disease.

Our study has several limitations. First, participants of the UKBB tend to be healthier, leaner, and smoke less compared to the general population of the UK, especially for those who accepted the invitation to take part in the accelerometer aspect of the study. Furthermore, UKBB participants were recruited when aged 40 to 69 years old, and so may now have an even higher risk of severe COVID-19 disease. The lower obese/overweight and smoking rate and older age may result in an underestimation of the effects of obesity and smoking status. Second, the data for AMPA were obtained between 2013 and 2015, and older age related to a lower AMPA, which could lead to an underestimation of the true effect of AMPA on COVID-19 [22]. The data for MVPA and measures of obesity were acquired even earlier (2006-2010), so, the historical measures may not be a good proxy for current values. Combining the discrepancy between objectively and subjectively measured PA and the time gap may explain the different results of MVPA and AMPA on COVID-19 related outcomes. Third, the definition of COVID-19 severity group is not perfect, the potential contamination among each group may bias our results. Finally, testing practices and capacity changed over time in the UK, so the earlier data (data before 30/05/2020) represent a limited subset of COVID-19 infections. Due to the limited number of test results, the MR analyses had low power to detect a significant association.

## CONCLUSION

This study supports a protective effect of objectively measured PA on COVID-19 outcomes after adjusting for age, sex, measures of obesity, and smoking status. Associations tend to be observed in patients with relatively mild symptoms (outpatient COVID-19 and overall COVID-19 instead of inpatient COVID-19 and COVID-19 death). These results suggest that physically active people may have a lower chance to be diagnosed with COVID-19 in general. Although the MR analyses did not support a causal association, that may be due to limited power. In this study, we conclude that policies to encourage and facilitate exercise at a population level during the pandemic should be considered.

## Additional material

Online Supplementary Document
