# Supplementary material for: Physical activity and COVID-19: an observational and Mendelian randomisation study
Source: J Glob Health. 2020 Dec 6;10(2):020514. doi: 10.7189/jogh-10-020514 (PMC7719276; doi:10.7189/jogh-10-020514)
Supplement: Online Supplementary Document [file jogh-10-020514-s001.pdf]

Title: Physical activity and COVID-19: an observational and Mendelian randomisation study

Authors: **Xiaomeng Zhang[1], Xue Li[1,5], Ziwen Sun[6,7], Yazhou He[1,2], Wei Xu[1], Harry Campbell[1], Malcolm G Dunlop[2], Maria Timofeeva[2,3*], Evropi Theodoratou[1,4*]**

[1]Centre for Global Health, Usher Institute, The University of Edinburgh, Edinburgh, United Kingdom

[2]Colon Cancer Genetics Group, Cancer Research UK Edinburgh Centre and Medical Research Council Human Genetics Unit, Medical Research Council Institute of Genetics and Molecular Medicine, The University of Edinburgh, Edinburgh, United Kingdom

[3]DIAS, Danish Institute for Advanced Study, Department of Public Health, University of Southern Denmark, Odense, Denmark

[4]Cancer Research UK Edinburgh Centre, MRC Institute of Genetics and Molecular Medicine, The University of Edinburgh, Edinburgh, United Kingdom

[5]School of Public Health and the Second Affiliated Hospital, Zhejiang University, Hangzhou, China

[6]Edinburgh School of Architecture and Landscape Architecture, The University of Edinburgh, Edinburgh, United Kingdom

[7]School of Design and Arts, Beijing Institute of Technology, Beijing, China

*: Corresponding authors

Evropi Theodoratou, Centre for Global Health, Usher Institute, University of Edinburgh, Edinburgh,

United Kingdom, e.theodoratou@ed.ac.uk, (+44) 0131 650 3210

Maria Timofeeva, Colon Cancer Genetics Group, Institute of Genetics and Molecular Medicine, University of Edinburgh, Edinburgh, United Kingdom, maria.timofeeva@igmm.ed.ac.uk, (+44) 131 651 8687

Supplementary methods

Self-reported moderate-to-vigorous PA (MVPA) data were mainly acquired during 2006 to 2010 through touch screen questionnaire [1] and acceleration vector magnitude PA (AMPA) data were collected from a subset of 103,687 UKBB participants wearing an accelerometer for 7 days between 2013 and 2015 [2]. For MVPA, we assigned as "NA" for individuals who selected "prefer not to answer" or "do not know" on the questions, individuals reporting being unable to walk, and individuals reporting MPA or VPA for more than 16 hours per day. We recoded those reporting >3 hours/day of MPA or VPA to 3 hours. MVPA was calculated by taking the sum of total minutes per week of MPA multiplied by four and the total number of VPA minutes per week multiplied by eight [3]. The AMPA covered 103,687 participants in UKBB, we removed participants whose data could not be calibrated, values that were unrealistically high (average vector magnitude > 100 mg), or who had poor wear-time. Measures of obesity (i.e. Body mass index (BMI), waist circumference and hip circumference) data were collected from 2006 to 2010 and BMI were calculated using manual measured weight and height by trained recruiters [4].

Table S1 Correlation coefficients between measures of obesity

	Body mass index	Waist circumference	Hip circumference
Body mass index	1		
Waist circumference	0.04*	1	
Hip circumference	0.06*	0.04*	1

*: P value<<0.001

Table S2 Sensitivity analysis for the association between physical activity measures and four COVID-19 related outcomes after excluding controls not from England, that died before 01/01/2020, and participants that tested negative for SARS-CoV-2

	Overall COVID-19				Inpatient COVID-19				Outpatient-COVID-19				COVID-19 death			
	MVPA (Subjectively measured)		AMPA (Objectively measured)		MVPA (Subjectively measured)		AMPA (Objectively measured)		MVPA (Subjectively measured)		AMPA (Objectively measured)		MVPA (Subjectively measured)		AMPA (Objectively measured)	
	OR (95%CI)	P	OR (95%CI)	P	OR (95%CI)	P	OR (95%CI)	P	OR (95%CI)	P	OR (95%CI)	P	OR (95%CI)	P	OR (95%CI)	P
Physical activity	1.01 (0.97, 1.06)	0.603	**0.83 (0.72, 0.96)**	**0.012**	1.05 (0.99, 1.11)	0.108	**0.82 (0.67, 0.99)**	**0.039**	0.94 (0.86, 1.02)	0.151	0.91 (0.72, 1.15)	0.423	1.03 (0.94, 1.14)	0.501	**0.54 (0.36, 0.81)**	**0.003**
Physical activity	1.01 (0.97, 1.06)	0.606	**0.79 (0.68, 0.91)**	**0.002**	1.05 (0.99, 1.11)	0.108	0.83 (0.68, 1.01)	0.065	0.94 (0.86, 1.02)	0.149	**0.74 (0.58, 0.94)**	**0.014**	1.03 (0.94, 1.14)	0.497	0.76 (0.51, 1.15)	0.191
Age	1.00 (1.00, 1.02)	0.002	0.97 (0.95, 0.99)	5.42E-04	1.02 (1.01, 1.03)	8.97E-07	1.00 (0.97, 1.02)	0.85	0.97 (0.96, 0.98)	1.55E-10	0.90 (0.88, 0.93)	2.26E-11	1.14 (1.12, 1.16)	4.32E-50	1.18 (1.10, 1.27)	1.75E-06
Sex	1.39 (1.27, 1.53)	5.87E-12	1.38 (1.06, 1.81)	0.019	1.57 (1.38, 1.77)	1.29E-12	1.85 (1.29, 2.66)	0.001	1.05 (0.89, 1.24)	0.535	0.85 (0.54, 1.34)	0.487	2.12 (1.73, 2.60)	5.86E-13	1.90 (0.96, 3.76)	0.067
Physical activity	1.02 (0.97, 1.06)	0.515	**0.79 (0.68, 0.92)**	**0.002**	1.05 (0.99, 1.11)	0.11	0.83 (0.68, 1.02)	0.073	0.95 (0.87, 1.03)	0.23	**0.74 (0.58, 0.95)**	**0.017**	1.04 (0.94, 1.14)	0.46	0.76 (0.51, 1.14)	0.189
Age	1.01 (1.00, 1.02)	0.002	0.97 (0.95, 0.99)	4.68E-04	1.02 (1.01, 1.03)	1.10E-06	1.00 (0.97, 1.02)	0.852	0.97 (0.96, 0.98)	1.34E-10	0.90 (0.87, 0.93)	1.18E-11	1.14 (1.12, 1.16)	4.31E-50	1.18 (1.10, 1.27)	1.78E-06
Sex	1.39 (1.26, 1.52)	1.52E-11	1.35 (1.03, 1.77)	0.03	1.56 (1.38, 1.77)	2.51E-12	1.82 (1.27, 2.62)	0.001	1.04 (0.88, 1.23)	0.625	0.81 (0.51, 1.29)	0.37	2.10 (1.71, 2.58)	1.30E-12	1.89 (0.95, 3.75)	0.068
Waist circumference	1.00 (1.00, 1.01)	0.221	1.00 (0.99, 1.01)	0.571	1.00 (1.00, 1.01)	0.048	1.01 (1.00, 1.03)	0.054	1.00 (0.99, 1.01)	0.813	0.99 (0.97, 1.00)	0.099	1.00 (0.99, 1.01)	0.797	1.00 (0.98, 1.03)	0.902
Hip circumference	1.00 (0.99, 1.00)	0.179	1.00 (0.99, 1.02)	0.856	0.99 (0.99, 1.00)	0.107	1.01 (0.98, 1.02)	0.863	1.00 (0.99, 1.01)	0.992	1.00 (0.97, 1.02)	0.904	1.00 (0.99, 1.01)	0.692	0.99 (0.95, 1.03)	0.66
BMI	1.01 (1.00, 1.01)	0.308	1.01 (0.98, 1.04)	0.532	1.00 (0.99, 1.02)	0.665	1.00 (0.96, 1.04)	0.96	1.01 (0.99, 1.03)	0.258	1.03 (0.98, 1.07)	0.209	1.01 (0.99, 1.03)	0.511	0.99 (0.92, 1.06)	0.744
Physical activity	1.00 (0.95, 1.06)	0.904	**0.81 (0.68, 0.95)**	**0.009**	1.05 (0.98, 1.11)	0.175	0.84 (0.67, 1.04)	0.102	0.93 (0.85, 1.03)	0.151	0.79 (0.60, 1.02)	0.072	1.00 (0.90, 1.11)	0.995	0.72 (0.46, 1.11)	0.135
Age	1.00 (1.00, 1.02)	0.002	0.98 (0.96, 1.00)	0.015	1.02 (1.01, 1.03)	2.09E-06	1.01 (0.98, 1.03)	0.568	0.97 (0.96, 0.98)	6.22E-10	0.90 (0.87, 0.93)	1.32E-09	1.14 (1.12, 1.17)	3.24E-44	1.21 (1.12, 1.31)	9.50E-07
Sex	1.41 (1.27, 1.56)	6.67E-11	1.42 (1.06, 1.90)	0.017	1.56 (1.37, 1.79)	8.10E-11	1.97 (1.33, 2.92)	0.001	1.12 (0.93, 1.33)	0.227	0.85 (0.52, 1.40)	0.52	2.01 (1.62, 2.51)	4.92E-10	1.62 (0.80, 3.27)	0.18
Waist circumference	1.00 (1.00, 1.01)	0.084	1.01 (1.00, 1.02)	0.143	1.01 (1.00, 1.01)	0.013	1.01 (1.00, 1.03)	0.037	1.00 (0.99, 1.01)	0.757	1.00 (0.98, 1.02)	0.731	1.00 (0.99, 1.01)	0.573	1.00 (0.98, 1.03)	0.944

Hip circumference	1.00 (0.99, 1.00)	0.143	1.00 (0.98, 1.01)	0.797	0.99 (0.99, 1.00)	0.083	1.00 (0.98, 1.02)	0.777	1.00 (0.99, 1.01)	0.936	0.98 (0.96, 1.01)	0.242	1.00 (0.99, 1.01)	0.977	1.00 (0.97, 1.04)	0.809
BMI	1.00 (0.99, 1.01)	0.445	1.01 (0.98, 1.04)	0.384	1.00 (0.99, 1.02)	0.758	1.01 (0.98, 1.05)	0.473	1.01 (0.99, 1.03)	0.478	1.01 (0.97, 1.07)	0.565	1.01 (0.98, 1.03)	0.608	1.01 (0.94, 1.08)	0.877
Smoking status																
Never (Reference)	1	/	1	/	1	/	1	/	1	/	1	/	1	/	1	/
Previous	1.28 (1.15, 1.44)	1.16E-5	1.40 (1.03, 1.91)	0.032	1.41 (1.22, 1.63)	3.09E-6	1.59 (1.06, 2.40)	0.025	1.01 (0.83, 1.24)	0.895	0.98 (0.57, 1.71)	0.953	1.64 (1.29, 2.08)	4.75E-5	1.93 (0.91, 4.12)	0.089
Current	1.37 (1.16, 1.62)	2.10E-4	1.82 (1.13, 2.94)	0.014	1.36 (1.08, 1.70)	0.008	2.03 (1.07, 3.85)	0.030	1.09 (0.82, 1.45)	0.566	1.51 (0.70, 3.25)	0.291	2.60 (1.87, 3.61)	1.34E-8	3.37 (1.06, 10.73)	0.040
Exposure to smoking at home	1.00 (0.98, 1.01)	0.505	0.95 (0.85, 1.05)	0.299	0.99 (0.97, 1.01)	0.366	0.95 (0.83, 1.09)	0.432	1.00 (0.99, 1.02)	0.66	0.96 (0.83, 1.11)	0.563	1.00 (0.97, 1.02)	0.878	0.64 (0.28, 1.46)	0.289
Exposure to smoking out of home	1.00 (0.98, 1.02)	0.928	0.91 (0.79, 1.05)	0.203	1.00 (0.97, 1.03)	0.979	0.90 (0.73, 1.10)	0.287	1.00 (0.97, 1.04)	0.793	0.80 (0.57, 1.11)	0.174	1.01 (0.97, 1.05)	0.656	1.00 (0.87, 1.15)	0.974

COVID-19: Coronavirus Disease 2019, MVPA: self-reported moderate-to-vigorous physical activity, AMPA: acceleration vector magnitude physical activity, BMI: body mass index, OR: odds ratio, CI: confidence interval

Table S3 Effect estimates of each instrumental variable of each exposure (MVPA, AMPA, BMI) on the overall COVID-19 outcome

SNP	Exposure	BP	CHR	EA	EAF	Beta.PA	SE.PA	Beta.BMI	SE.BMI	Beta.COVID	SE.COVID
rs149943	MVPA	28002388	6	G/A	0.85	0.019	0.005	-0.004	0.006	-0.067	0.059
rs2035562	MVPA	85056521	3	A/G	0.33	-0.014	0.004	-0.011	0.004	-0.021	0.046
rs2854277	MVPA	32628084	6	C/T	0.92	0.031	0.008	-0.003	0.005	-0.079	0.097
rs2988004	MVPA	37044388	9	T/G	0.56	-0.013	0.003	-0.010	0.004	0.036	0.044
rs3094622	MVPA	30327952	6	A/G	0.86	0.02	0.005	-0.003	0.005	0.009	0.063
rs7791992	MVPA	50237784	7	C/A	0.41	-0.014	0.003	0.005	0.004	-0.075	0.044
rs7804463	MVPA	133447651	7	T/C	0.53	0.015	0.003	-0.001	0.004	-0.068	0.043
rs59499656	AMPA	40768309	18	A/T	0.655	-0.028	0.005	0.011	0.004	-0.062	0.045
rs6895232	AMPA	152039421	5	T/A	0.663	0.027	0.005	0.009	0.009	0.009	0.046
rs564819152	AMPA	21820650	10	A/G	0.679	0.028	0.005	-0.013	0.004	0.056	0.047
rs55657917	AMPA	44326864	17	A/G	0.77	-0.037	0.005	-0.004	0.005	0.106	0.054
rs6775319	AMPA	18758501	3	A/T	0.271	0.027	0.005	-0.009	0.004	0.02	0.049

SNP: single-nucleotide polymorphism, EA: effect allele, EAF: effect allele frequency, PA: physical activity, SE: standard error, COVID: Coronavirus Disease 2019, MVPA: self-reported moderate-to-vigorous physical activity, AMPA: acceleration vector magnitude physical activity, BMI: body mass index

1 Sudlow C, Gallacher J, Allen N, Beral V, Burton P, Danesh J, et al. UK biobank: an open access resource for identifying the causes of a wide range of complex diseases of middle and old age. PLoS Med 2015; 12:e1001779.

2 Doherty A, Jackson D, Hammerla N, Plotz T, Olivier P, Granat MH, et al. Large Scale Population Assessment of Physical Activity Using Wrist Worn Accelerometers: The UK Biobank Study. PLoS One 2017; 12:e0169649.
3 Klimentidis YC, Raichlen DA, Bea J, Garcia DO, Wineinger NE, Mandarino LJ, et al. Genome-wide association study of habitual physical activity in over 377,000 UK Biobank participants identifies multiple variants including CADM2 and APOE. Int J Obes (Lond) 2018; 42:1161-76.
4 Bycroft C, Freeman C, Petkova D, Band G, Elliott LT, Sharp K, et al. The UK Biobank resource with deep phenotyping and genomic data. Nature 2018; 562:203-9.
